# Mapping the Antimicrobial Supply Chain in Bangladesh: A Scoping-Review-Based Ecological Assessment Approach

**DOI:** 10.9745/GHSP-D-20-00502

**Published:** 2021-09-30

**Authors:** E.S.F. Orubu, M.A. Samad, M.T. Rahman, M.H. Zaman, V.J. Wirtz

**Affiliations:** a Department of Biomedical Engineering, Boston University College of Engineering, Boston, MA, USA.; b Institute for Health System Innovation & Policy, Boston University Boston, MA, USA.; c Antimicrobial Resistance Action Centre, Bangladesh Livestock Research Institute, Savar, Dhaka, Bangladesh.; d Department of Microbiology and Hygiene, Faculty of Veterinary Science, Bangladesh Agricultural University, Mymensingh, Bangladesh.; e Department of Global Health, Boston University School of Public Health, Boston, MA, USA.

## Abstract

**Introduction:**

Maintaining access to antimicrobials while preventing misuse is essential to combating the threat of antimicrobial resistance (AMR). The study objectives are to propose a framework of 16 indicators that can be used at the national level to assess the capacity to ensure access and curtail inappropriate use and to profile the antimicrobial supply chain for Bangladesh.

**Methods:**

Using a framework based on a rational construct, we assessed the antimicrobial supply chain of Bangladesh, with a focus on key players and products using a scoping review to obtain and describe information on 16 indicators. With players, we mapped linkages, manufacturers’ production capacity, and ownership, among others, and demand point characteristics—pharmacy and pharmacist density, pharmacy/medicine outlets dispersion, veterinary clinic/hospitals, veterinarians’ density, product quality, and regulation. We assessed product characteristics including listing on the World Health Organization (WHO) Model Essential Medicines List (EML) and WHO Access, Watch, and Reserve (AWaRe) classification of the major (top 10) antibiotics for human use; the proportion of medically important antimicrobials (MIAs) in veterinary use; and pricing. Production capacity and price controls were used to assess access and listing on the WHO EML, AWaRe/MIA classification, and a calculated pharmacy-to-pharmacist ratio to assess use.

**Results:**

Bangladesh has a high (98%) local antibiotic production capacity with pricing controls indicating the ability to ensure access. The presence of a high proportion of medicine outlets not under the control of pharmacists (4:1) and the high percentages of WHO Watch (54%) and MIAs (90%) of the major antibiotics are indicators of possible misuse.

**Discussion:**

Most of the data used in the framework were publicly available. Bangladesh has the capacity to ensure access but needs to strengthen its ability to regulate the quality of antimicrobials and prevent their inappropriate use through antimicrobial stewardship at the community (medicine outlet) levels to check AMR. There may also be a need for more regulations on licensing of MIAs.

## Abstract (precis)

A standardized method for evaluating antimicrobial supply chains in the context of access and use could be a useful tool in assessing national capacity to implement programs that address antimicrobial resistance. We present both a novel ecological approach comprising mapping and the use of indicators that can be used to characterize national antimicrobial supply chains as well as benchmark countries and, for the first time, a country-level assessment of Bangladesh.

## Teaser

**Key Messages:**

- This framework proposes a novel method for antimicrobial medicines supply chain mapping using 16 indicators with a focus on access and use in the context of antimicrobial resistance containment.
- Countries, especially low- and middle-income countries, can apply this framework to rapidly assess supply chain gaps and to identify areas for targeted interventions on access and use of antimicrobials.
- Bangladesh’s antimicrobial supply chain was profiled, identifying strengths in ensuring access to antimicrobials and gaps in use and regulation.

**Key Implications:**

- Policy makers, program managers, and Antimicrobial Resistance Containment One-Health Secretariat committees could use this framework to perform a rapid situational analysis (strength, weakness, opportunities, and threats) of the antimicrobial/antibiotic supply chain for human and veterinary medicines at the national level to identify target areas for intervention. This can include policy interventions via restrictions on licensing of certain antibiotics of critical importance to humans, as Bangladesh is doing with colistin. Through an analysis of the brief market dynamics presented here, program managers can make assessments of supply risks.
- Regulators could use the mapping to identify geographical   regions with high pharmacy densities for more targeted inspections for medicines quality assurance.
- One-Health Secretariat committees could use professional   densities numbers to advocate for more training and capacity building for pharmacists and veterinarians.

## INTRODUCTION

Antimicrobial resistance (AMR)—the ability of microorganisms to withstand treatment with therapeutic doses of an antimicrobial agent—is a public health emergency requiring concerted global multisectoral strategies aimed at containment through curbing inappropriate use while ensuring access.^1^^–^^3^ The inappropriate use of antimicrobials includes practices such as self-medication, sale of antimicrobials without prescription, noncompliance with prescribed doses, prescribing without reference to sensitivity tests, use of substandard and falsified antimicrobials, and nontherapeutic uses for growth promotion or prophylaxis of medically important antimicrobials (MIAs) in livestock and contributes to AMR in public health.^4^^–^^6^ Access to medicines within health systems is a 5-dimensional concept comprising availability, affordability, geographical accessibility, acceptability or rational selection and use, and quality.^7^ Balancing access with use means that populations get the antimicrobials they need (evidence-based prescribing), when they need them (appropriate use), at the right price, at the right place, and of the right quality. A holistic assessment of national antimicrobial supply chains can help simultaneously identify challenges with access and use.

Maintaining access to antimicrobials through the pharmaceutical supply chain—the network of players and processes through which medical products move from the source to the end-user—needs to be balanced against inappropriate use. For most countries, there are guidelines for medicine use, including formularies and standard treatment guidelines. The World Health Organization (WHO) maintains the Model Essential Medicines List (EML) as a guide for the appropriate selection and use of medicines by nations.^8^ Additionally, it introduced the Access, Watch, and Reserve (AWaRe) categorization, which groups antibiotics according to their potential to induce resistance and clinical utility. With veterinary uses, measures to reduce AMR in public health include restrictions in the use of MIAs, which are antimicrobials used in human health, and reduction in the overall use of all antimicrobials.^9^^,^^10^ These guidelines specify institutional controls on the use of antimicrobials in all sectors as strategies to combat AMR.

Maintaining access to antimicrobials through the pharmaceutical supply chain needs to be balanced against inappropriate use.

A well-developed pharmaceutical supply chain ensures access. In a well-regulated system, the demand points for medicines—the distribution point to the end-user within formal and informal health systems—are manned by professionals with the skill set to ensure supply while preventing misuse or abuse, thus contributing toward balancing access and use.

Bangladesh is a densely populated South Asian country identified as one of several with a high risk for AMR.^11^ A systematic review of AMR in Bangladesh conducted in 2018 reveals, for example, that organisms causing urinary tract infections showed high levels of resistance to penicillins (ampicillin and amoxiclav), ranging from 58%–100%.^12^ Against the cephalosporins (cefotaxime, ceftazidime, and ceftriaxone), the most commonly used class of antibiotics, resistance was similarly high at ≥55% in *E. coli* and ≥78% in *Klebsiella* spp.^12^^–^^14^ Comparative WHO GLASS 2019 data shows *E. coli* isolated from urine samples resistant to ciprofloxacin in 11.5% of patients (n=82,2931) in the United Kingdom to 89.7% (n=394 patients) in Bangladesh.^15^ Against ceftriaxone, *E. coli* resistance was 10.1% (n=87,398) in the United Kingdom and 63.9% (n=394) in Bangladesh.^15^

Bangladesh runs a pluralistic health system with 5 parallel systems of medicine: allopathic, ayurvedic, herbal, homeopathic, and *unani.*^16^ Regulatory oversight of all 5 systems is provided by the National Medicine Regulatory Agency, the Directorate General of Drug Administration (DGDA).^17^ Antimicrobials, classified under the allopathic category, constitute a significant size of the domestic pharmaceutical market. In 2016, antimicrobials (systemic anti-infectives) ranked second in sale volumes (18%) after medicines for alimentary and metabolism or the gastrointestinal tract (36%).^18^ The total market size for pharmaceuticals sale was estimated at about US$2.5 billion in 2018.^19^

There are 3 main pathways for the supply of allopathic medicines in Bangladesh: formal public, formal private, and informal private sector.^20^ In terms of intended use and financing, the market for antimicrobials for human use can be structured into 3: antimicrobials for TB, antimicrobials used for maternal and child health, and all other antimicrobials that are licensed by the DGDA and available for sale through both formal and informal private channels. Antimicrobials for TB and maternal and child health are largely supplied for free to the end-user through public and private not-for-profit facilities under the formal public channel with the participation of key development partners and nongovernmental organizations.^20^ In addition, outside of these antimicrobials for these specific conditions and cases, the government provides free public health care at all levels from specialized facilities at the tertiary level in administrative divisions to community clinics to make health care geographically accessible. However, utilization is low, with most people (67%) seeking health care, including medicines, in the private sector.^16^

Although there are several studies addressing challenges with access, misuse, quality, AMR, or regulatory governance in LMICs,^21^^–^^24^ there is as yet no standard method for analyzing or mapping antimicrobial supply chains that addresses both access (including medicines quality) and inappropriate use. There is an urban health atlas mapping health facilities in Bangladesh.^25^ However, there is no current comprehensive national-level assessment of the formal and informal antimicrobial supply chain to characterize challenges with the dynamics of access, use, medicine quality, and regulatory governance for the human and livestock sectors in Bangladesh.

There is as yet no standard method for analyzing or mapping antimicrobial supply chains that addresses both access and inappropriate use.

As part of a broader One-Health project assessing behavioral, practice, and policy factors contributing to the indiscriminate uses of antimicrobials, including poor-quality medicines, and AMR, in Bangladesh, we have evaluated the National Action Plans on AMR containment in Bangladesh and 7 other LMICs and assessed the integrity of the antimicrobial supply chain in Bangladesh.^26^ This study complements this previous body of work. This present study aimed to map the supply chain for human and veterinary antimicrobials, with a focus on the implications for public health. The objectives were to: (1) propose a novel method for antimicrobial supply chain analysis to identify challenges with access and use in the context of AMR, and (2) apply the development to describe/characterize the supply chain for antimicrobials in Bangladesh. While the scope is One-Health, comprising humans and animals, in recognition of the impact of irrational uses of antimicrobials in animals as a driver of AMR, the article focuses on public health implications, in essence on human health.

## METHODS

This study employed a qualitative ecological design with a scoping review methodology to profile the antimicrobial supply chain in Bangladesh. We adopted a 5-tier model of pharmaceutical supply chains consisting of primary manufacturer of active pharmaceutical ingredient (API) (Tier 1), secondary manufacturer of finished pharmaceutical product (FPP) (Tier 2), main distributor (Tier 3), local distributor (Tier 4), and demand point (Tier 5).^27^ The scoping review methodology was adopted to enable comprehensive data collection, considering that some of the information sought on these players and processes in the supply chain may not be available as peer-reviewed literature.

We performed an ecological assessment of the supply chain using a constructed framework of 16 selected indicators to characterize: (1) the antimicrobial supply chain, (2) manufacturers, (3) sales and dispensing, or demand, points (4) regulation, and (5) licensed antimicrobial products (Table 1).

**TABLE 1.** Framework^a^ of Selected Indicators Used to Map the Antibiotic Supply Chain in Bangladesh

	**Indicator**		
**Element**	**Characteristic**	**Metric(s)**	**Rationale/Assumption**
Supply chain	Linkages	Schematic	Maps product flow from manufacture to use
Manufacturers	Market structure	Market share of the top 10 by value (concentration)	For identification and classification
	Ownership	% local/domestic	Local production supports access^b^ (availability)
	Overall production capacity	% supplied locally	
	Source of active ingredients	% sourced locally	
	Specialization in antimicrobial production	Proportion of manufacturers producing the top 10 by volume (%); high/low	Low specialization can protect against shortages and guarantee availability but may be a risk for drug quality
Regulation^c^	Technical capacity	WHO classification of NRA	A WHO Stringent Regulatory Authority of Maturity Level 3 or 4 is a competent NRA able to ensure medicines quality in the supply chain
Demand points	Pharmacy density	No./ 5 km^2^ and No./10,000 population	Quantification and dispersion measures of (geographic) access; poor surveillance of a large number of pharmacies can impede product quality
	Pharmacist density	No./10, 000 population	A measure of professional capacity to guard against potential misuse
	Veterinary clinic density	No./ 5 km^2^	Geographical accessibility to licensed demand points promotes rational use
	Veterinarian density	No./10,000 population	A measure of professional capacity to guard against potential misuse
	Product quality	Prevalence of SF medicines	A measure of the integrity of the supply chain
Antimicrobial products with market authorization (license)	WHO AWaRe categories	%	High % of Watch and Reserve groups may indicate (potential) misuse
	Medically important antibiotics	%	
	Listing of top 10 in EML	%	Proxy for rational selection and use–the acceptability component of access
	Pricing/price controls	Present/absent	Proxy for access (affordability). High prices may promote misuse through poor adherence

Abbreviations: AWaRe, Access Watch and Reserve classification; EML, Essential Medicines List; NRA, National Regulatory Authority; SF, substandard and falsified medicines; WHO, World Health Organization.

^a^ This framework is not intended to be exhaustive.

^b^ Access is described here using the 5-dimensional framework of Wirtz et al.,^7^ consisting of availability, affordability, geographical accessibility, acceptability, and quality.

^c^ Regulation is an overarching feature of the supply chain, not 1 distinct element.

To obtain these indicators, we performed a scoping review for information, first for the general pharmaceutical supply chain, and then specifically for the antimicrobial supply chain as summarized here and detailed in the Supplement.

### Literature and Database Review for Information on the General Pharmaceutical Supply Chain

To scope the general pharmaceutical supply chain in Bangladesh, a search was conducted on 4 databases: Banglajol (a service that provides access to Bangladesh Online Journals), Google Scholar, PubMed, and the DGDA database.

## RESULTS

The medicine supply chain in Bangladesh, based on data from the DGDA dashboard, is complex (Table 2). Overall, for products, there are 43,529 registered drugs from 761 manufacturers distributed through 120,871 retail outlets in the country. In terms of players, for allopathic drugs alone, there are 118,519 wholesale and retail outlets as of March 2020.

**TABLE 2.** Characteristics of Supply Chain Elements for All 5 Medicine Systems in Bangladesh

	**Allopathic**	**Ayurvedic**	**Homeopathic**	**Herbal**	**Unani**	**Totals**
						
Registered drugs	29,813	4,119	2,417	550	6,630	43,529
Generics^a^	3,642	N/A	N/A	N/A	N/A	N/A
Manufacturers	204	202	42	35	278	761
Wholesale pharmacies	1,165	N/A	N/A	N/A	N/A	N/A
Retail outlets	117,354	409	2411	11	686	120,871

Abbreviation: N/A, not applicable or not available.

^a^ Generic is defined as an unbranded product.

Source: Directorate General of Drug Administration dashboard (as of February 16, 2020).

Specific supply chain elements are presented and discussed in the following sections.

### Antimicrobial Supply Chain

**FIGURE 1.**
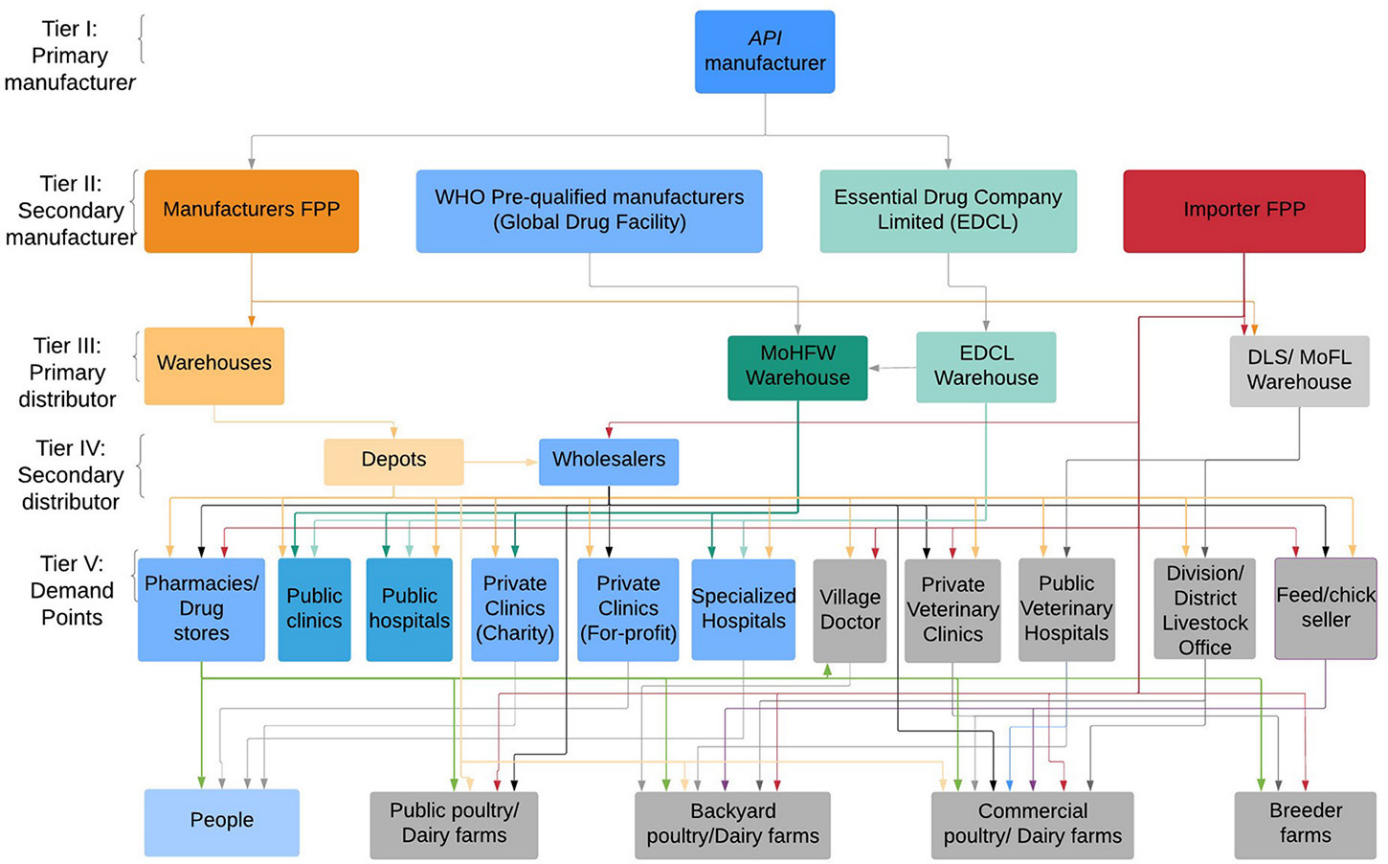
Schematic of the Network Structure of the Antimicrobial Supply Chain for Human and (Terrestrial) Animal Sectors in Bangladesh Illustrating the Flow of Product^28^^–^^30^^,^^34^^–^^46^^a^ Abbreviations: DLS, Department of Livestock Services; EDCL, Essential Drug Company Limited of Bangladesh; MOHFW, Ministry of Health and Family Welfare, Bangladesh; MOFL, Ministry of Fisheries and Livestock. ^a^ At the lower tiers, blue represents the human sector. In general, gray represents the animal sector exclusively, apart from the village doctor. The *palli chikitshak/pallachikitos* or “village doctor” (someone without a medical or MBBS degree) is a term applied to various persons including the “pharmacist,” someone who is regarded as knowledgeable to provide advice and medicines for human and veterinary use, sometimes a licensed medical professional who does locum in a pharmacy/drug store in a “doctor’s chamber” attached to this facility, or a cadre of trained informal health care providers, albeit without authorization to prescribe/dispense antimicrobials outside of the list of over-the-counter drugs. The specialized hospitals represent a mix of public and private hospitals providing advanced care, including TB. Data sources for the animal sector were supplemented by expert knowledge provided by Md. Samad and Tanvir Rahman.

Figure 1 maps the supply chain network for human and animal use antimicrobials in Bangladesh.

Bangladesh imports APIs for its pharmaceutical industry. Almost all APIs (reported at between 97%–99.5%) are imported from Tier I manufacturers, based mainly in China (40%), India (30%), and Korea (10%),^31^ as well as in Vietnam, Europe, United States, and Japan.^19^^,^^32^ There is only limited domestic API production in 4 pharmaceutical firms: Square, Beximco, Globe, and Gonoshasthaya Pharmaceuticals Limited. There are plans for the establishment of a local API industry in Munshiganj district, 40 km southeast of Dhaka.^33^

There are 3 key player types in the antimicrobial supply chain at Tier II in Bangladesh with different distribution networks and processes: private sector manufacturers and importers; the Global Drug Facility; and the public Essential Drug Company Limited (EDCL). In the for-profit private channel, 78 manufacturers produce antimicrobials for human use, and 44 produce antimicrobials for veterinary use (with overlaps). The supply of TB, HIV, and malaria medicines (antimicrobials) is controlled by international development partners – the Global Drug Facility – and the government of Bangladesh. Procurement is facilitated by the development partners, storage by the government, and distribution by the government through its facilities. Thus, this system is more regulated. While the EDCL produces and supplies all government facilities, private manufacturers also supply these public health facilities through tenders. The EDCL produces 27 antimicrobials.^34^ Importers of FPP are mainly nonpharmaceutical companies who largely procure medicines intended for veterinary uses. These firms typically also supply animal feed (medicated) and other products for use in the agricultural sector or other industries.

Generally, the key manufacturers in the private sector own their distribution channels (Tiers III and IV). All top 10 manufacturers of human antimicrobials maintain distribution networks, each with about 16–33 depots, including warehouses, throughout the country for a total of **231** distribution centers for 9 manufacturers; we could find no information about 1 manufacturer.^35^^–^^42^ Distribution networks are either an integral part of the manufacturer or subsidiary specialized distribution sister companies. The Ministry of Health and Family Welfare maintains a central medical store as its warehouse, as does the Ministry of Fisheries and Livestock; and EDCL has warehouses at its production facilities. Most wholesalers are located in markets in Dhaka, with the Mitford market hosting the greatest numbers.^43^ All manufacturers maintain direct marketing teams to canvas from demand points all over the country.

There are 11 demand points (Tier V) through which people and animals obtain medicines, comprising retail outlets, private and public health care facilities, and sale points for animal medicines including the village doctors (*pallachikitos)*, a feature of the rural health care system. The number of retail outlets (private medicine outlets) is estimated to be about twice the registered drug outlets, or about 200,000.^44^ The District Health Information System 2 (DHIS2) health facility registry for Bangladesh lists 23,926 public and private facilities^45^; almost all of which dispense drugs, including antimicrobials, and, thus, under the ambit of the DGDA.

Table 3 summarizes other characteristics of the antimicrobial supply chain, as presented in-depth in the following sections.

**TABLE 3.** Indicators for Key Elements of the Antimicrobial Supply Chain in Bangladesh Based on the Constructed Framework

	**Indicator**		
	**Characteristic**	**Metric(s)**	**Value**
**Supply chain**	Linkages	Schematic	N/A
**Manufacturers**	Market structure	Top 10 market share by value	≈70%
		CR_4_	255, human sector
			178, animal sector
	Ownership	Domestic	90%
	Overall production capacity	Supplied locally	98%
	Source of active ingredients	Local	≤3%
	Specialization in antimicrobial production	Proportion of manufacturers producing the top 10 by volume	38–63%, human sector
			25–84%, animal sector
**Demand points**	Pharmacy density	No./5 km^2^	<1–7^a^
		No./10,000 population	7.2
	Pharmacist density	No./10,000 population	1.8
	Veterinary clinics/hospitals density	No.	428 public veterinary hospitals
	Veterinarian density	No./1,000,000 livestock	≈1–2
	Product quality	Prevalence of SF medicines	0.04%
**Regulation**	Technical capacity	WHO classification of NMRA	Not a stringent regulatory authority
**Antimicrobial products with market authorization (license)**	WHO AWaRe category “Watch”	Top 10 human use	54%
	Medically important antibiotics	Top 10 (animal use)	90%
	Listing of top 10 in EML	Top 10 (human use)	62%
	Pricing/price controls	Present/absent	Present

Abbreviations: CR_4_, four firm concentration ratio; EML, Essential Medicines List; N/A, not applicable; NMRA, National Medicines Regulatory Authority; SF, standard formulary; WHO, World Health Organization.

^a^ Depending on the district, and based on only 30%, or 46,161 currently licensed outlets at the time of the study of 117, 354.

### Manufacturers

**FIGURE 2.**
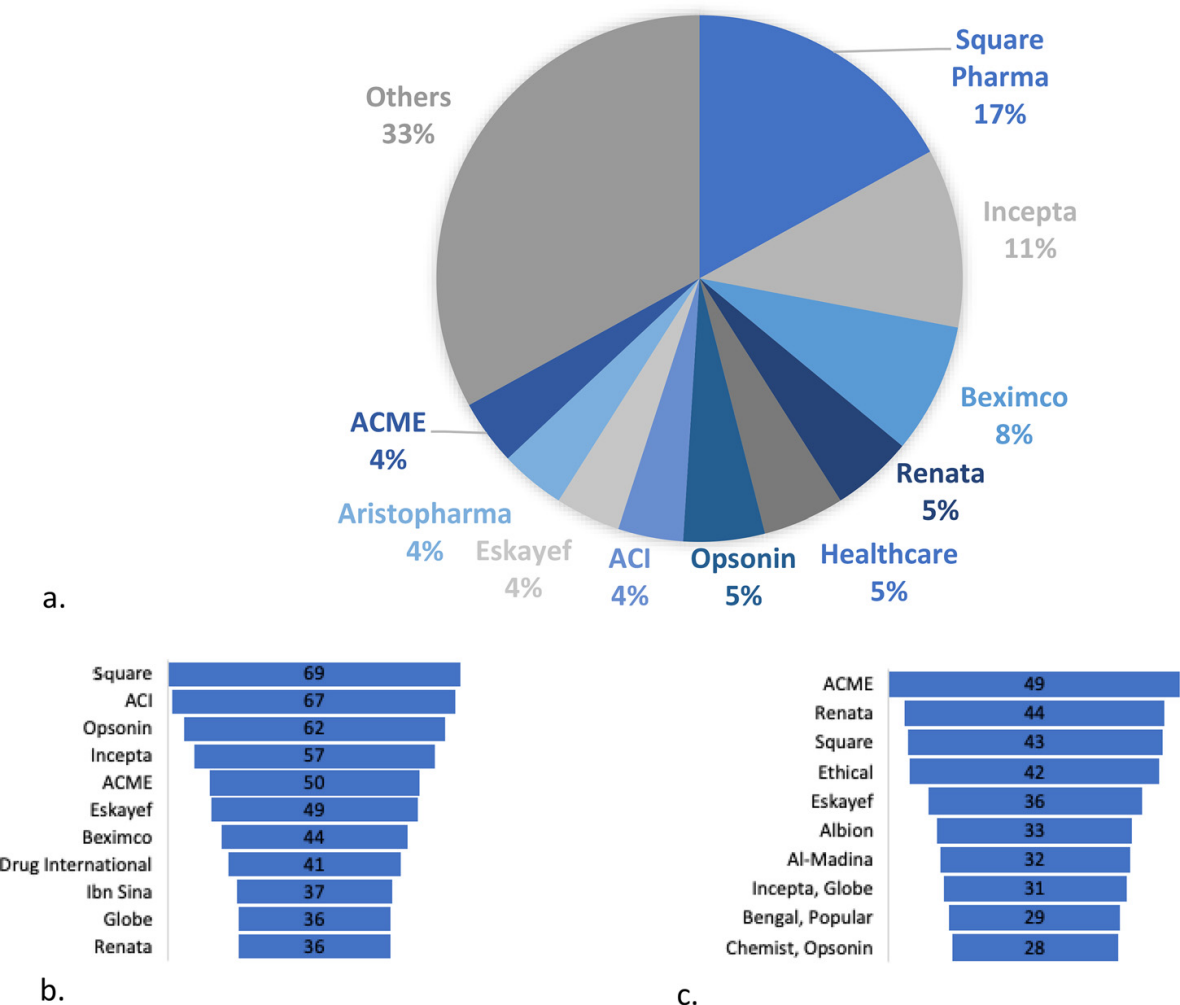
(a) The top 10 pharmaceutical manufacturers by market share in value in Bangladesh, 2018. Top 10 human antimicrobial producers, by proportion of licensed INNs, for: (b) human antimicrobials, (c) animal antimicrobials in Bangladesh^a^ ^a^ There was a greater similarity between the top 10 manufacturers by market share and the 10 producers of human antimicrobials (73%, 8/11) than with the top 10 producers of animal antimicrobials (46%, 6/13). With human antimicrobials, producers not among the top 10 by value were: Drug International, Ibn Sina and Globe. With animal antimicrobials, these were: Ethical, Albion, Al-Madina, Globe, Bengal, Popular and Chemist. In either sector, human or animal, the difference between market shares by value and volumes suggest diversification or branding among manufacturers.

**Market structure**: Overall, the pharmaceutical market is protected and dominated by domestic brands. Pharmaceutical manufacturing is concentrated, with 10 of the registered 204 allopathic manufacturers controlling about 70% of the market and 3 controlling one-third by market share (Figure 2). The top 10 are Square, Incepta, Beximco, Renata, Healthcare, Opsonin, ACI, Eskayef, Aristopharma, and ACME. Of these 10, Square has been the market leader for more than 3 decades in terms of sales value, with a market share of 18% in 2018.^46^

The ranking of manufacturers by the number of licensed antimicrobials for human and veterinary use produced reveals a sort of diversification or branding strategy. While most are also in the top 10 by sales values, several of these (ACI, Opsonin, ACME, and Eskayef) produce more antimicrobials by volume, even though their share of the market is only about 4%–5% by sales (Figure 2).

The market for antimicrobial production is oligopolistic, as indicated by a Concentration Ratio of the 4 largest antimicrobial producing firms greater than 60, or CR_4_ > 60 (Table 3).

**Ownership**: Manufacturers are mostly (90%) domestic, following divestments by multinational pharmaceutical companies consequent on regulatory changes instituted in the 1980s, with one of the last, Sanofi, scheduled to leave by 2020 and under buy-out consideration by Beximco as of January 2021.^47^^,^^48^

**Production capacity:** Bangladesh produces almost all (97%) of its medicines locally, mostly as generics.^19^

**Specialization:** There is low specialization among manufacturers of antimicrobials with high contributions to the overall market. More than 10 different manufacturers produce each of the major antimicrobials for veterinary use, ranging from 11–37 (25%–84%, n=44); for antimicrobials for human use, this is between 30–49 (38%–63%, n=78). In both sectors, there is a correlation between the number of licensed formulations and the number of producers.

### Demand Point Characteristics

**Pharmacy dispersion**: There is 0.9 retail outlet/drug store per km^2^ (117,354 registered allopathic pharmacies on a landmass of 130,170 km^2^).

**FIGURE 3.**
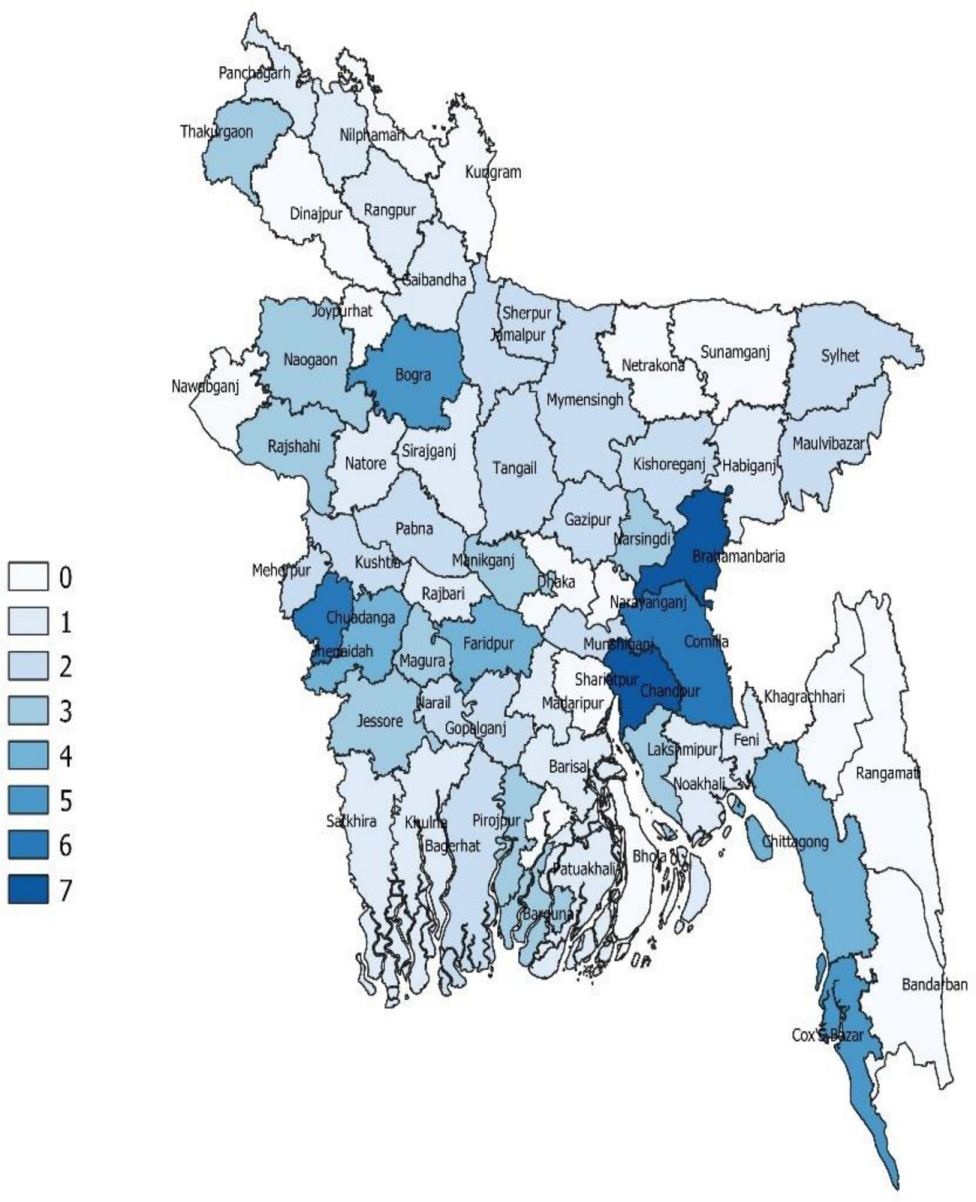
District-Wise Distribution of Retail Pharmacy Outlets in 64 Districts of Bangladesh^a^ ^a^The numbers are number of pharmacies/medicine outlets per 5 km^2^. Numbers refer to currently valid licensed outlets per district as obtained from the DGDA Registration Dashboard as at May 12, 2020. Each of the seven intervals signify a range. For example, 0 indicates 0–1, 1 indicates 1–2 pharmacies per 5 km^2^, etc. Thus, 0 does not necessarily imply the absence of a pharmacy/medicine outlet but means that the pharmacies are more dispersed, or fewer in overall numbers, except for the two districts of Lalmonirhat and Sunamganj, where there did not seem to be any currently licensed pharmacy/medicine outlet, with the Directorate General of Drug Administration database showing license expired for all listed premises, as at the time the study was conducted. Nawabganj is the same as Chapai Nawabganj.

The subregional (district) distribution of licensed retail outlets, based on the currently licensed total of 46,161 as of May 2020, ranges from <1 up to 7 per 5 km^2^ across the 64 districts (Figure 3).

**FIGURE 4.**
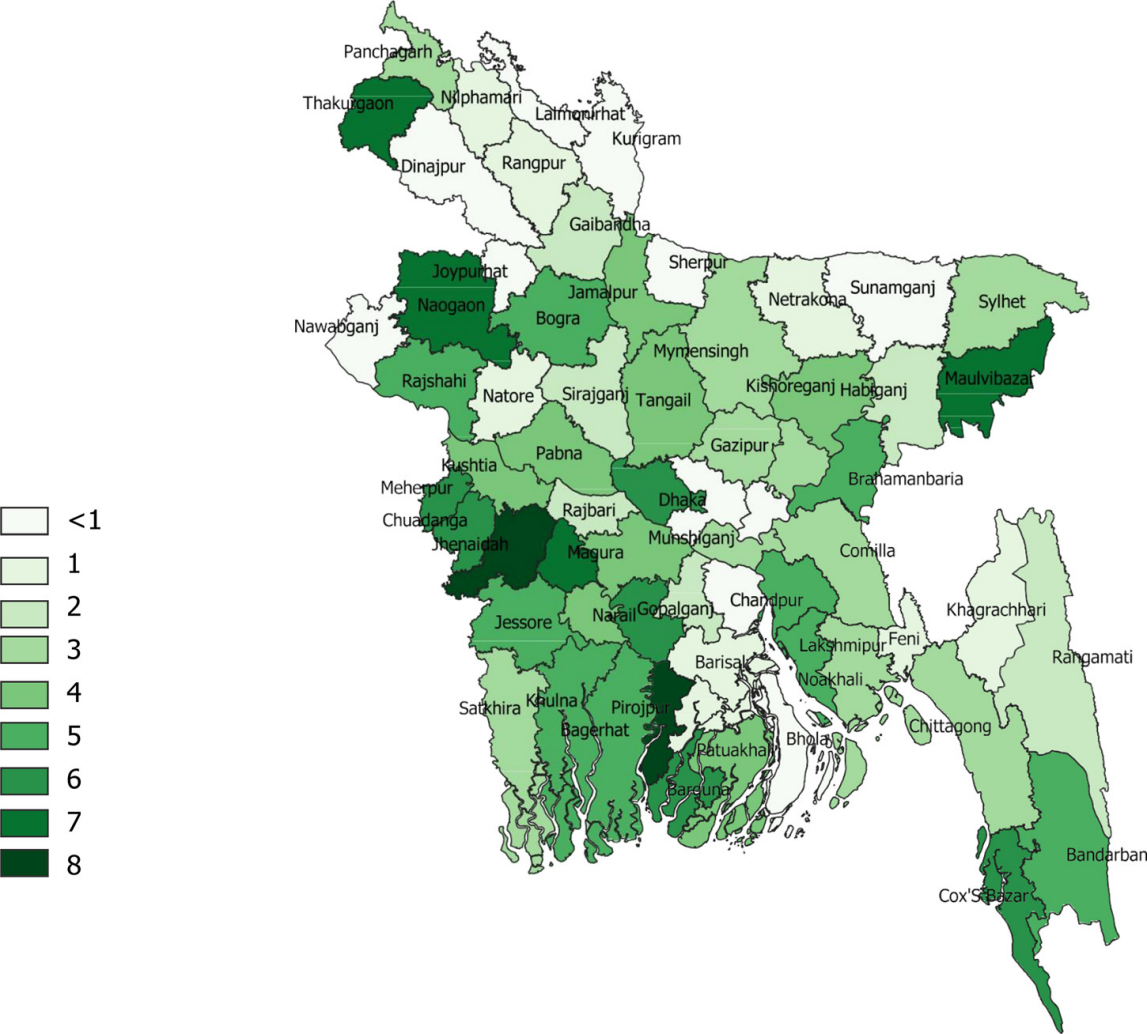
Pharmacy Density Per 10,000 Population at the District Level in Bangladesh^a^ ^a^Numbers represent ranges. For example, 1 is a nominal 1 to <2 per 10,000 population, similar to numbers in Figure 3, with <1 suggesting an overall fewer registered pharmacy/medicine outlets per 10,000 population, as at study completion in May 2020.

**Pharmacy density**: There are 7.2 retail outlets per 10,000 population (based on 117,354 registered pharmacies for 162 million people). The regional distribution is shown in Figure 4.

**Pharmacist density**: There are 1.8 pharmacists per 10,000 population.^49^ Thus, the pharmacy to pharmacist ratio is 7.2/1.8, or 4:1.

**Veterinary clinics**: There is 1 public veterinary hospital at each *upazilla* (subdistrict) or county-level with 481 as at 2015.^50^

**Veterinarians’ density**: 1-2 per 0.7 million livestock.^51^

**Medicines quality**: 0.04% prevalence of substandard and/or falsified medicines in 2011.^52^

**Regulatory Capacity**: The DGDA is currently not regarded as a stringent regulatory authority.^53^

### Licensed Antimicrobial Products

#### Antimicrobials for Use in Human Health

There are 138 unique, or individual, licensed antimicrobials/anti-infectives by international non-proprietary name for human health commercially available as 1,763 products in the Bangladesh National Formulary 2019; the majority (76%, 1,345/1,763) of which are antibiotics. The antibiotics belong to 8 antibiotic classes, with 4 constituting 92% of all antibiotic products in the market (n=1,345).

The most common antibiotic class was cephalosporin (44%, 597/1,345); followed by penicillin (18%, 236/1,345); quinolone/fluoroquinolone (15%, 203/1,345); and macrolide (15%, 200/1,345). The other antimicrobial classes are: carbapenem and monobactam (2%), tetracycline (2%), sulfonamide (2%), and aminoglycoside (1%).

With almost all licensed antimicrobials (99.5%) locally manufactured, imported products were few, with only 8 products, representing 0.5% of all licensed antimicrobial products, imported. For 5 of the imports, there were also domestic alternatives.

The top 10 antimicrobials ranked by number of licensed/commercial formulations are shown in Table 4. These 10 constitute three-quarters (75%, 1,003/1,345) of all licensed antibiotics. Among the 10, cephalosporin was the most common antibiotic class with 4 members (cephradine-cilastatin, cefuroxime, cefixime, and cefpodoxime) constituting more than one third or 37.5% (376/1,003); followed by macrolide (azithromycin and erythromycin) (17%, 171/1,003); penicillin (amoxicillin and flucloxacillin), (16%, 162/1,003); and the fluoroquinolones, ciprofloxacin, and levofloxacin (13%, 133/1,003).

**TABLE 4.** AWaRe Classification and Listing of the Top 10 Antimicrobials for Human Use by Total Licensed Formulations

**Rank**	**INN**	**Total Licensed Formulations**	**WHO (EML)**	**Pakistan (EML)**	**India (NF)**	**AwaRe Class **
1	Azithromycin	116	Yes	Yes	Yes	Watch
	Cephradine-cilastatin	116				Access
2	Cefuroxime	99	Yes	Yes		Watch
3	Cefixime	97	Yes	Yes	Yes	Watch
4	Ciprofloxacin	96	Yes	Yes	Yes	Watch
5	Amoxicillin	84	Yes	Yes	Yes	Access
6	Flucloxacillin	78				Access
7	Nitazoxanide	64				N/A
	Cefpodoxime	64				Watch
8	Metronidazole	60	Yes	Yes	Yes	Access
9	Erythromycin	55		Yes	Yes	Watch
10	Levofloxacin	37	Yes	Yes		Watch
	Cotrimoxazole	37	Yes	Yes	Yes	Access

Abbreviations: AWaRe, Access, Watch, and Reserve classification; EML, Essential Medicines List; INN, international nonproprietary names of pharmaceuticals; N/A, not applicable or not classified; NF, National Formulary; WHO, World Health Organization.

**AWaRe categories**: Most (54%, 7/13) of the top 10 antimicrobials belong to the WHO Watch category, with 39% in the Access category (Table 4). Nitazoxanide, used for the treatment of infective enteritis caused by giardia, is an uncategorized antimicrobial.

**Listing:** Only 62% (8/13) of the top 10 antibiotics were contained in the WHO EML (Table 4). This is comparable to India, with a 54% (7/13) similarity, and Pakistan at 69% (9/13). Flucloxacillin, cephradine-cilastatin, nitazoxanide, and cefpodoxime were not listed in any of these compendia.

**Pricing**: There are price controls with ceilings for all antimicrobials. Prices are published in the compendium.

#### Antimicrobials for Use in Animal Health

Of a total of 1,338 products licensed for veterinary use on the DGDA allopathic medicine database (as of March 26, 2020), 61% or 818 were antimicrobials. These were 73 unique licensed antimicrobials consisting of 30 MIAs, which were commercially available as 428 formulations; 32 nonmedically important antimicrobials in 235 formulations; and 11 anthelmintics as 155 formulations. Antibiotics as a percentage of total antimicrobials for animal use was 85% (62/73). Similarly, MIAs as a percentage of total antimicrobials for animal use was 41% (30/73).

With licensed antimicrobials manufactured locally, imports were few or absent. For example, there were no listed imported ceftriaxone or oxytetracycline, and only 10 listed ciprofloxacin products imported from manufacturers in Korea. The antimicrobials imported included unique combinations not manufactured locally, for example, ampicillin+colistin, amoxicillin+colistin, amoxicillin+erythromycin+colistin, and amoxicillin+gentamycin. These products were imported mostly (85%, 11/13) from Korea; then China, and the Netherlands (1 each).

The most common antibiotic classes, comprising over half (55%) of all licensed antibiotics, were: fluoroquinolone (18%, 119/663); tetracycline (17%, 112/663); penicillin (10%, 64/663); and sulfonamide (10%, 64/663).

All but 1 (90%) of the top 10 antibiotics licensed for animal use in Bangladesh were medically important (Table 5).

**TABLE 5.** Selected Characteristics of the Top 10 Antibiotics Licensed for Veterinary Use by Number of Products in Bangladesh

**Rank**	**INN**		**No. Licensed Products**	**Antibiotic Class**	**Medically Important **
**1**	Oxytetracycline		98	Tetracycline	No
**2**	Ciprofloxacin		67	Fluoroquinolone	Yes
**3**	Amoxicillin		47	Aminopenicillin/penicillin	Yes
**4**	Metronidazole		28	Nitroimidazoles	Yes
**5**	Gentamycin		27	Aminoglycoside	Yes
**6**	Ceftriaxone		24	Cephalosporin	Yes
**7**	Sulphamethoxazole + trimethoprim		23	Sulfonamides	Yes
**8**	Doxycycline		14	Tetracycline	Yes
**9**	Neomycin		12	Aminoglycoside	Yes
**10**	Benzyl penicillin + procaine penicillin		11	Penicillin	Yes

Abbreviation: INN, international nonproprietary names of pharmaceuticals.

## DISCUSSION

This ecological assessment profiles the antimicrobial supply chain in Bangladesh for the first time to the best of our knowledge using a novel methodology comprising mapping and the use of indicators to describe access and use in the context of AMR.

The methodology proposed in this study can be used for the comparative evaluation of LMICs antimicrobial supply chains to understand specific challenges to target under national action plans on containing AMR, especially as proxy measures for access and use as contained under strategic objective number 4 in the WHO Global Action Plan on antimicrobial resistance containment.^3^ Of the 8 goals listed under this objective, this article covers 6, or 75%, excluding diagnostic laboratories and antimicrobial stewardship considerations. There is no standardized method or approach to antimicrobial supply chain analyses, thereby, preventing any attempt at comparative evaluations. Previous studies in the field have included a variety of methods and reporting styles, informed by study objectives.^21^^–^^24^ The framework proposed in this study can be applied in the comparative analysis of countries with similar contextual challenges such as in, for example, the WHO South East Asia region.

The methodology proposed in this study can be used for the comparative evaluation of LMICs antimicrobial supply chains to understand specific challenges to target under national action plans on containing AMR.

Bangladesh has the capacity for the local manufacturing and distribution of antimicrobials/medicines, suggesting both commercial availability and geographical access. This capacity sets Bangladesh apart from other LMICs in the same low-income grouping, in terms of local production as a means of improving availability.^54^^,^^55^ The pharmaceutical industry is the second highest foreign exchange earner for Bangladesh; exporting to more than 100 countries.^56^ In 2015, Bangladesh was granted an exemption under the TRIPS agreement up to 2023, or until graduation from the Least Developed Country status, thus ensuring that its pharmaceutical industry can produce generics or branded generics without regard to patent protection.^57^ The concentration of manufacturing among a few suggests consolidation of the pharmaceutical manufacturing industry as an option to improving quality. Interestingly, Bangladesh has a public company manufacturing essential medicines. This is unusual for many countries and could serve as a model for other LMICs.

### Regulatory Capacity Challenge

Overall, the medicine supply chain in Bangladesh is complex, with implications for effective regulatory oversight, appropriate use, and medicine quality. The DGDA regulates all 5 medicine systems (allopathic, ayurvedic, homeopathic, herbal, and *unani*) with the mandate to control medicine quality, set prices, inspect all premises, and perform postmarketing surveillance. To be able to fulfill all these functions, the DGDA capacity needs to be increased. This is particularly important both in light of the WHO’s finding that poor-quality medicines tend to proliferate in contexts where low regulatory capacity coexists with high demand for medicines and low affordability, and the impact of poor-quality medicines on AMR.^58^ A focused study of the DGDA conducted in 2015 highlighted some challenges it faces, including human capacity and infrastructural,^20^ in agreement with the WHO Classification as a nonstringent regulatory agency.^53^

### Sales or Dispensing Points Complexity

The high complexity of Tier V (demand points) is a large challenge for effective surveillance and inspection. This has several implications. The pharmacy density in Bangladesh is higher than in many other countries or regions. For example, at 7.2/10,000 population, this is triple the density in the USA at 2.2/10,000 population^59^^,^^60^; double the average density for the European Union, with mean pharmacy density of 3.1/10,000 population; and slightly more than India’s with an estimated 5.5/10,000 population in 2018.^61^^,^^62^ The sheer number of small outlets will require a large human workforce of inspectors to ensure adequate supervision including of good storage and pharmacy practices for antimicrobials. For instance, in a nationally representative study of drug stores, only a third (n=111) had a functional refrigerator required for the proper storage of temperature-sensitive drugs.^60^ However, the relatively low spatial distribution (about 1/km^2^) of licensed medicine outlets, suggesting clustering, implies that most could be easily visited for regulatory inspections.

The high complexity of Tier V (demand points) is a large challenge for effective surveillance and inspection.

Drug stores outnumber formal providers, illustrating a similar gap in most LMICs.^63^ The distribution of retail outlets shows a rural-urban divide. It is estimated that 58% of the drug stores/pharmacies are in the old Dhaka and Chattogram Divisions, both of which contribute to over half of the population of Bangladesh, and are mostly urban areas.^64^ Public health facilities are more nationally distributed with a presence at all administrative divisions. For example, community clinics are available at the lowest level of the society; and 1 livestock clinic per *upazilla* or sub-district level.^16^ The exact numbers of the *pallachikitos*—a feature of the rural health care system—are unknown. Although antimicrobials are allopathic, all 5 medicine systems in Bangladesh have been known to prescribe/dispense antimicrobials. Controlling use/demand at all these demand points is a second challenge and would require a lot of effort.

There is a shortage of professional capacity (pharmacists) in Bangladesh, with drug stores manned by nonprofessional staff. This gives rise to the third challenge of how to staff this large number of sale points with trained personnel to ensure Good Dispensing and Pharmacy Practices. An estimated pharmacist density of 1.8 per 10,000 population means that there are only about 29,160 pharmacists for Bangladesh’s 162 million people, insufficient to man the over 100,000 registered medicine outlets.^49^ In this, Bangladesh is similar to Nigeria (1.3/10,000), and Pakistan (1.6/10,000), but different from India with about 9/10,000.^65^ There are 3 classes/grades of persons who can sell medicines in Bangladesh, differentiated on the length of “training” into Grades A, B, and C. Grade A are equivalent to pharmacists in other settings and are those with a University Degree in Pharmacy. Grade B hold a 1-year diploma and may be the equivalent of Pharmacy Technicians. The lowest class, Grade C, requires only an apprenticeship lasting only a few weeks (3–4 months). Sale and dispensing points are characterized by the near-complete absence of Grade A pharmacists, consisting majorly of Grade C “pharmacists.”^66^ This gap would take time to fill. This is a fundamental weakness in the pharmaceutical supply chain, as evidenced by reports from other countries in the global south where medicine outlets manned by nonprofessionals are significant sources of substandard and falsified medicines.^67^^,^^68^ With this pharmacy density, Bangladesh is among the bulk of LMICs with less than 5 pharmacists per 10,000 (where this implies, by default, a 1:1 pharmacist: pharmacy ratio), implying a shortage of professional capacity, or a surplus of drug stores.^49^

In the veterinary sector, the 2015 Performance of Veterinary Services Gap Analysis Report for Bangladesh recommends a significant increase in the number of veterinarians over the next 5 years to an estimated 2,130 for the public sector—suggesting a human resource gap in this sector. There is no comprehensive information for the private sector.

Placing all veterinary uses of antimicrobials under a veterinarian is a means of ensuring quality use. In Bangladesh, medicines for use in animals are obtained from a variety of demand points not under the control of a trained veterinarian.^69^^,^^70^

In terms of medicine quality, we did not find recent estimates from the DGDA. The reported estimate is from 2011. In contrast, India publishes periodic reports on the substandard and falsified medicines withdrawn from the market.^71^ Alternative data sources could be reports from scientific or medicine quality surveys.

Bangladesh has initiated several moves to improve sales and dispensing practices of antimicrobials. By law, antimicrobials are prescription-only medicines, with only metronidazole included in the over-the-counter list for drug stores. However, this policy is, by and large, not adhered to, not only in Bangladesh but also in other LMICs.^21^^,^^23^ Recently, it has adapted the Accredited Drug Dispensing Outlet initiative first implemented in Tanzania to create 2-tiers of pharmacies—a model pharmacy staffed by a trained pharmacist, and a lower-level medicine shop without the requirement to be manned by a pharmacist.^72^ These are obligated to sell antibiotics only on a prescription basis. However, this concept is not without its challenges.^73^^–^^75^ Furthermore, there is the plan to mandate antimicrobials to be packaged in red packs—both to create public awareness and to prevent misuse.^76^

### Antimicrobial Products

The high proportion of licensed WHO Watch antibiotics has implications for AMR development and spread. Of the 4 antibiotic classes that make up the bulk of the licensed antibiotic products in Bangladesh, over half belong to the Watch group, including all macrolides and fluoroquinolones and the majority of cephalosporins. This high level of market offering of Watch antibiotics as opposed to the Access group has been observed also in India and Pakistan.^77^^,^^78^ The target of the AWaRe categorization is 3-fold: achieve an increase in the use of Access antibiotics, the group associated with the lowest potential to induce AMR, to 60% by 2023; decrease the use of Watch antibiotics; and restrict Reserve antibiotics to infections caused by drug-resistant infections.^14^ This target may be challenging in contexts with a large proportion of licensed antibiotic products being of the Watch category and with unrestricted access via over-the-counter sales of antibiotics, with implications for antimicrobial stewardship programs.^78^

The high proportion of licensed WHO Watch antibiotics has implications for AMR development and spread.

Similarly disturbing is the extremely high proportion of MIAs which means that a large proportion of licensed antimicrobials for animal use belong also to the human therapeutic arsenal.^9^ The usage of these antimicrobials has been correlated with AMR in poultry in neighboring India, for example.^79^ The WHO recommends the restriction of all non-therapeutic uses of MIAs for growth promotion or disease prevention in animals. Bangladesh has made recent progress in this area with the de-licensing of most colistin formulations—an MIA ranked as critically important, but we still found combinations containing colistin being imported. This calls for further actions to restrict access. There may also be a need for more regulations on licensing of other MIAs including critically important antibiotics such as gentamycin, neomycin, ceftriaxone, and ciprofloxacin.

### Limitations

One limitation with the proposed framework is that it does not assess use by the conventional methods such as antimicrobial consumption and point-prevalence surveys which provide standardized measures of inappropriate use. However, these data are unavailable for many LMICs, among other reasons because acquiring primary data via surveillance and point-prevalence studies requires considerable human resources. The indicators assessed provide proxy measures for characterizing antimicrobial supply chains to highlight challenges. We also did not consider in depth the medicine quality aspect of access here as this was outside the scope of this work.

## CONCLUSION

This work presents a novel method for performing supply chain analyses. Using data from Bangladesh as a model LMIC, it maps the supply chain for antimicrobials used in the human and animal sectors in the context of antimicrobial resistance containment to highlight gaps for targeted interventions. In so doing, it also presents, to the best of the authors’ knowledge, the first mapping of the supply chain for Bangladesh. This framework can be used to map the antimicrobial supply chain in LMICs.

It represents a lean method of analysis that can supplement ongoing efforts by development, national, and international health authorities to address the urgent threat of AMR. The 16 data types it includes are relatively easy to collect, making the method useful for countries with human capacity and technology constraints to rapidly assess deficits in the supply chain for antimicrobials. Importantly, the proposed method incorporates salient market/economic features such as market structure, specialization, and pricing—often underappreciated or neglected as important components of supply chain analyses. The framework, thus, presents a holistic and efficient tool for antimicrobial/antibiotic supply chain analysis.

Specifically, the methodology proposed in this study, the data collected in the study, and information on the challenges in collecting data not included in the analysis can be used to better inform decisions about interventions (including communities to engage, whether regulatory, private sector, or others) to address the emergence and spread of AMR.

## Supplementary Material

20-00502-Orubu-Supplement.pdf

## Notes

**Peer Reviewed**

**First published online:** August 25, 2021.
