# Supplementary material for: Mapping the Antimicrobial Supply Chain in Bangladesh: A Scoping-Review-Based Ecological Assessment Approach
Source: Glob Health Sci Pract. 2021 Sep 30;9(3):532–47. doi: 10.9745/GHSP-D-20-00502 (PMC8514039; doi:10.9745/GHSP-D-20-00502)
Supplement: 20-00502-Orubu-Supplement.pdf [file 20-00502-Orubu-Supplement.pdf]

Supplement to: Orubu ESF, Samad MA, Rahman MT, Zaman MH, Wirtz VJ. Mapping the antimicrobial supply chain in Bangladesh: A scoping-review based ecological assessment approach. *Glob Health Sci Pract*. 2021;9(3). https://doi.org/10.9745/GHSP-D-20-00502

To obtain indicators, we performed a scoping review for information, first for the general pharmaceutical supply chain, and then specifically for the antimicrobial supply chain.

These four databases were searched for information describing any aspect – players or processes – of the 5-tiers model of the pharmaceutical supply chain in Bangladesh. Search terms were: "medicine supply chain", "pharmaceutical supply chain" and "drug supply chain" on Banglajol; and "Bangladesh medicine supply chain" and "Bangladesh pharmaceutical supply chain" on Google Scholar and PubMed. On PubMed the equivalent MeSH Terms were: equipment and supplies; medicine; Bangladesh with the subheading: supply and distribution. On Banglajol and PubMed, titles containing any of the search terms were retrieved for inclusion in the Zotero Reference Manager software. Included abstracts were then screened for eligibility. Abstracts with no mention of any aspect of the pharmaceutical supply chain were excluded. With Google Scholar, titles with supply chain in association with medicine or pharmaceutical(s) OR with pharmaceutical (with or without Bangladesh) but without supply chain were selected for inclusion in the reference manager, Zotero. Titles with supply chain but with livestock (poultry, fish, shrimp), or agriculture without medicine or pharmaceutical were excluded. The included articles were then reviewed. Studies in locations other than Bangladesh and studies in Bangladesh that did not describe the pharmaceutical supply chain were excluded. The literature search was performed on March 25, 2020.

Data collected included information on the general distribution network of medicines in Bangladesh, or linkages, the top 10 manufacturers in terms of market share, and demand point characteristics.

The **DGDA** medicines dashboard was searched on February 16, 2020 for the number of licensed manufacturers, importers, wholesalers and retail outlets/demand points for all medicines [27].

Document, database and websites review to map the supply chain for antimicrobials

To map the supply chain for antimicrobials, we performed a review of national compendia, manufacturers' websites and databases to identify all licensed antimicrobials, manufacturers, and the distribution network of key manufacturers as described:

Products (licensed antimicrobials): Human sector: The national compendium, the Bangladesh National Formulary (BNDF) 5th edition, 2019, was manually searched for licensed antimicrobials for human use. All medicines listed under anti-infectives were entered into an Excel spreadsheet. Data extracted were: the International Non-proprietary Name (INN), or generic name; the total number of formulations for each and all listed INN; the producers of the different formulations; and the proportion of total listed INN produced by each manufacturer. The top 10 antimicrobials by number of licensed formulations were identified. A formulation, or product, is defined as one

discrete dosage form type, for example ampicillin 500 mg capsule and ampicillin 250 mg capsule are two different formulations of ampicillin. Imported medicines were noted. The search was conducted in March, 2020.

Veterinary antimicrobials: The DGDA database for allopathic medicines was searched for antimicrobials licensed for veterinary use [27]. The complete database of human and veterinary medicines was downloaded on March 26, 2020. Using filters, the total number of products listed in the database for each INN licensed either for human use or veterinary use was noted. Antimicrobials (antibacterial and anthelminthic medicines) for veterinary use were then extracted into an Excel spreadsheet. Antimicrobials were identified from practice experience, supplemented by a search on DrugBank™ (a publicly available online database of drug information) in case of doubt [28]. Antimicrobials for veterinary use were stratified into those also used in human medicines, or MIAs, and those used exclusively in animal medicine, or non-medically important. Antimicrobials used only in animals were identified as those with exclusive licensing status for veterinary use on DrugBank™. Combinations of substances used in animals only with antimicrobials licensed exclusively for use in humans were excluded. Non-antimicrobial substances were also excluded. All included antimicrobials were then listed by manufacturer.

Manufacturers: All manufacturers of these antimicrobials for human and animal uses extracted from the review of the compendium and DGDA database as described above were noted. Manufacturers were ranked by volume (number of antimicrobials licensed) to identify the top 10. The websites of the top 10 antibiotics producers for human medicine were then reviewed. Data collected were: the number of distribution channels (warehouses and depots); API production status; and ownership (whether domestic or foreign).

Sales or dispensing points: Five sales or dispensing point characteristics were assessed: pharmacy density, pharmacist density, pharmacy spatial distribution, veterinary clinic/hospital density and veterinarian density. The DGDA database was used to map pharmacy density in Bangladesh. The list of all licensed medicine outlets in the private sector, as at May 12, 2020, was reviewed and those with current licenses were counted for each of the 64 districts[27]. Current license means a valid license to operate as identified on the DGDA database. The WHO Global Health Observatory was assessed to obtain information on pharmacist density [29]. Information on veterinarians' density and number of veterinary clinics/hospitals was obtained from a literature review. The quality of medicines in circulation was assessed using pharmacovigilance reports by the DGDA [30] Regulatory capacities were identified from WHO and DGDA websites and or reports.

To map the public sector for human health, two other databases were searched: (i) the Essential Drug Company Limited (**EDCL**) website [31]; and (ii) the **eLMIS** (electronic Logistics Management Information System) dashboard of the District Health Information System 2 (DHIS2) maintained by the Ministry of Health and Family Welfare (MoHFW) in Bangladesh[32]. The EDCL website was

reviewed both for records of antimicrobials produced and facilities supplied. The supply chain for maternal and child health was described using information available on the eLMIS dashboard.

Analysis of results

As per our framework (Table 1), the antimicrobial supply chain (network) was mapped and presented as a schematic to describe linkages.

In evaluating market structure, the **key players** (major manufacturers) were **identified** and **ranked** in terms of market size both by share value and by volume, or proportion of licensed antimicrobials manufactured. To determine market concentration, we applied the four-firm concentration ratio (CR_4) using the total market size by volume of the four largest antimicrobials manufacturers where a value of <40% indicated competition; 40-60%, monopolistic competition and >60% meant an oligopoly. The major manufacturers were further **categorized** into: domestic-, or foreign-owned. To analyze specialization in antimicrobial production, the proportions (%) of manufacturers producing each of the top 10 antimicrobials were computed and categorized as high or low. To evaluate production capacity, we assessed imports as a proportion (%) of licensed products. Similarly, the sources of APIs were analyzed as proportions (%) procured by country.

To evaluate sales or dispending points, we used five **measures**: pharmacy density, pharmacist density, pharmacy dispersion index, veterinary clinic/hospital density, and veterinarian density [20]. Pharmacy/pharmacist density was defined as the number of pharmacies/pharmacists per 10,000 population. The pharmacy dispersion index, or spatial distribution, was defined as the number of retail outlets per 5 square kilometers of landmass, calculated using the number of currently licensed outlets at the time of the study which was 46,161. The population was taken as 162 million, and landmass as 130,170 square kilometers [33]. Pharmacy spatial dispersion was mapped and visualized using QGIS 3.18 Zurich – a Open-Source Geographic Information System – with geographic data from GADM [34,35]. The ratio of pharmacy to pharmacist density was used as a measure of professional capacity, with unity taken as the ideal. Pharmacy density and pharmacy dispersion were employed as measures of geographical access. Similar parameters were employed for the veterinary sector, with veterinarians' density expressed in terms of number of veterinarians per million livestock units. The quality of medicines was assessed as prevalence in percentage. Regulatory human capacity was expressed as number of regulatory staff to pharmacies/medicine outlets and regulatory technical capacity in terms of WHO Stringent Authority classification [36]

For licensed antimicrobial products, the top 10 antibiotics for human use were analyzed using the WHO AWaRe categorization [37] and benchmarked against purposively-selected regional and global comparators. The selected regional countries and the associated compendia were: India (National Formulary, NF, 2016), Pakistan (Essential Medicines List, EML, 2018); and the global comparator was the current edition of the WHO Model EML [8]. Similarly, using the WHO classification, the proportion (%) of the major (top 10) antibiotics for animal use that were MIA was assessed [9].

Supplement to: Orubu ESF, Samad MA, Rahman MT, Zaman MH, Wirtz VJ. Mapping the antimicrobial supply chain in Bangladesh: A scoping-review based ecological assessment approach. *Glob Health Sci Pract*. 2021;9(3). https://doi.org/10.9745/GHSP-D-20-00502
